# Efficacy of macrogol 4000 plus electrolytes in bowel preparation for colonoscopy in patients with chronic constipation

**DOI:** 10.1186/s12876-021-01976-2

**Published:** 2021-10-19

**Authors:** Ryoji Ichijima, Sho Suzuki, Mitsuru Esaki, Tomomi Sugita, Kanako Ogura, Chika Kusano, Hisatomo Ikehara, Takuji Gotoda

**Affiliations:** 1 Division of Gastroenterology and Hepatology, Department of Medicine, Nihon University School of Medicine, 1-6 Kanda-Surugadai, Chiyoda-ku, Tokyo, 101-8309 Japan; 2 Department of Medicine and Bioregulatory Science, Graduate School of Medicine Sciences, Kyushu University, Fukuoka, Japan; 3 Department of Gastroenterology, Internal Medicine, Kitasato University School of Medicine, Sagamihara, Japan

**Keywords:** Macrogol 4000, Colonoscopy, Chronic constipation, Polyethylene glycol, Efficacy, Safety

## Abstract

**Background:**

Chronic constipation is a significant factor in poor bowel preparation for colonoscopy. Macrogol 4000 plus electrolytes (Movicol, EA Pharma, Tokyo, Japan), containing polyethylene glycol (PEG) and electrolytes, have been used recently to treat patients with constipation. However, prospective studies on the use of macrogol 4000 for bowel cleansing for colonoscopy are lacking. This study aimed to investigate the efficacy and safety of macrogol 4000 in addition to PEG administered in patients with chronic constipation.

**Methods:**

This single-center, single-arm prospective study enrolled patients with chronic constipation who were scheduled to undergo colonoscopy. The primary endpoint was the proportion of good bowel preparation assessed using the Boston bowel preparation scale (BBPS) (6 or more points). The secondary endpoints were the time from when pPEG (MoviPrep, EA Pharma, Tokyo, Japan) was taken until colonoscopy could be started, amount of PEG taken, number of defecations, whether additional PEG doses were taken, and adverse events. Endoscopy-related endpoints included cecal intubation rate, insertion time, observation time, adenoma detection rate (ADR), and polyp detection rate (PDR). The tolerability of PEG and macrogol 4000 was assessed using a questionnaire.

**Results:**

Forty patients were included in the analysis. The median BBPS was 7 (range 3–9) and ≥ 6 points in 37 cases (92.5%). The median time until colonoscopy can be started was 210 min (90–360 min), the median volume of PEG taken was 1500 mL (1000–2000 mL), and the median number of defecations was 7 (3–20). No adverse events were observed. Fourteen patients required an additional dose of PEG. Cecal intubation was achieved in all cases, the median insertion time was 6.0 min (range 2.3–22 min), and the median observation time was 8.8 min (range 4.0–16.0 min). The ADR and PDR were 60.0% and 75.0%, respectively. A proportion of patients rated the tolerability of macrogol 4000 and PEG as 95.0% and 50.0%, respectively.

**Conclusions:**

Intake of macrogol 4000 in addition to PEG is effective and safe for colonoscopy in patients with chronic constipation.

*Clinical trial registration statement* This study was registered in the UMIN-CTR database (UMIN-ID000038315).

## Background

Colorectal cancer is the second most common cause of cancer-related deaths worldwide, and the number of patients is expected to continue to increase in the future [1]. Endoscopic polyp resection has been shown to be effective in preventing the development of colorectal cancer [2]. However, colonoscopy bowel preparation is reportedly ineffective in approximately 20–25% of cases [3]. Poor bowel cleanliness reduces the adenoma detection rate (ADR) and cecal intubation rate, prolongs observational time, and shortens the interval between colonoscopies, all of which have deleterious effects on patients [4–7]. The reported prevalence of chronic constipation in the general population varies between 2 and 27% [8]. Chronic constipation ranks alongside diabetes as a risk factor for ineffective bowel preparation for colonoscopy [9].

Polyethylene glycol (PEG) is currently the standard colonic cleansing agent used for colonoscopy. In Western countries, 4000 mL of PEG is used for colonic cleansing [10, 11]. However, 10–15% of patients are reportedly unable to tolerate this dose and do not complete bowel preparation [12]. Therefore, attempts have been made to reduce the amount of PEG taken. A number of randomized controlled trials have shown that a PEG dose of 2000 mL can provide results equivalent to those of PEG 4000 mL and is more tolerable than 4000 mL [13–15]. However, some patients have difficulty in tolerating even a dose of PEG 2000 mL. Hence, further efforts are required to improve bowel preparation decreasing the dose of PEG.

Macrogol 4000 plus electrolytes (Movicol, EA Pharma, Tokyo, Japan) is a non-stimulant laxative that has recently been approved for use in Japan [16]. Owing to its ability to dissolve in apple juice or sports drinks, it is better tolerated than PEG formulations. To the best of our knowledge, no prospective study on the use of macrogol 4000 as a bowel-cleansing agent prior to colonoscopy has been reported. Therefore, we investigated the efficacy and safety of the additional dose of macrogol 4000 to PEG in patients with chronic constipation as bowel preparation, administered the evening before colonoscopy.

## Methods

### Study design

This was a single-center prospective study. The subjects were patients with chronic constipation who were scheduled to undergo colonoscopy between November 2019 and July 2020. This study was registered in the UMIN-CTR database (UMIN-ID000038315).

### Inclusion and exclusion criteria

Patients with chronic constipation who met all the inclusion criteria and underwent colonoscopy were enrolled in the study. The definition of “constipation” was that of the 2016 revised Rome IV criteria, under which a patient must have experienced at least two of the following symptoms: (a) straining for > 25% of defecations, (b) lumpy or hard stools (form 1 or 2 on the Bristol Stool Form Scale) for > 25% of defecations, (c) sensation of incomplete evacuation for > 25% of defecations, (d) sensation of anorectal obstruction/blockage for > 25% of defecations, (e) manual maneuvers to facilitate defecation (e.g. digital evacuation, pelvic floor support) for > 25% of defecations, and (f) < 3 spontaneous bowel movements per week. They must also meet the criterion that loose stools are rarely present without the use of laxatives. The definition of “chronic” was that the patient had experienced symptoms for at least the past 6 months and had met the above criteria for the past 3 months. The inclusion criteria for this study were as follows: (1) patients with chronic constipation scheduled to undergo colonoscopy, (2) aged ≥ 20, and (3) provided consent to participate voluntarily and in writing, following a full explanation by an investigator. The exclusion criteria were as follows: (1) individuals with colorectal lesions undergoing colonoscopy for preoperative investigation or treatment, (2) previous gastrointestinal surgery (other than appendectomy or hemorrhoidectomy), (3) structural chronic constipation, (4) familial adenomatous polyposis, (5) inflammatory bowel disease, (6) severe renal dysfunction, (7) inability to ingest pretreatment for colonoscopy, (8) pregnancy or lactation in women, (9) allergy to the trial drug, (10) already using macrogol 4000, (11) inability to provide informed consent, or (12) considered unsuitable to participate in the study by the investigator. The patients could continue using regular laxatives during the study.

### Bowel preparation method and colonoscopy

The patients enrolled in this study received a low residual diet on the day before colonoscopy and four sachets (6.8 mg/sachet) of macrogol 4000 as a colonic cleanser at 20:00 on the same day. The four sachets of macrogol 4000 were dissolved in approximately 250 mL of water and taken at once. The liquid used for dissolving macrogol 4000 (apple juice or sports drink) was selected by the patient. The patients ingested 1500 mL of PEG formulation (Moviprep, EA Pharma, Tokyo, Japan) in our hospital’s colonoscopy room at 9:00 on the morning under supervised endoscopy. They ingested a cupful of the dose (~ 200 mL) for over about 15 min, followed by ingestion of half a cup of water. This was repeated until the defecation was clear. The nurse checked the patient's defecation, and when it became clear and colorless, the nurse determined that the colonoscopy could be started.

The following parameters were recorded: time taken from PEG intake until the nurse considered that colonoscopy could be started, dose of PEG used, number of defecations, and any adverse events. All patients received a PEG dose of 1500 mL even if the colonoscopy could be started when they had taken less than this amount. If the nurse considered that a patient was not ready for colonoscopy after taking a PEG dose of 1500 mL, more PEG was administered until colonoscopy became feasible. Colonoscopy was started after the patient had taken 2000 mL of PEG even if defecations had not become clean. All colonoscopies were conducted by doctors working in our hospital who were certified by the Japanese Gastroenterological Endoscopy Society. A PCFQ260AZI microscope (Olympus, Tokyo, Japan) was used. All the procedures were performed with CO_2_ insufflation. If the patient requested, the procedure was started under sedation with midazolam (1–3 mg), and this dose was increased by 1 mg, in case the patient experienced discomfort. If the endoscopists requested, the procedure was conducted using an antispasmodic, such as butyl scopolamine or glucagon. The patient’s blood pressure and respiratory state were monitored during each procedure.

### Study endpoints

The primary endpoint was the proportion of patients with good bowel preparation. Bowel preparation was assessed using the Boston bowel preparation scale (BBPS) [17].

The secondary endpoints were bowel preparation or endoscopy-related evaluation and tolerability of the agents. The bowel preparation endpoints were time taken from PEG ingestion until colonoscopy could be started, volume of PEG ingested, number of bowel defecations, whether additional doses were required, and adverse events. Endoscopy-related endpoints included cecal intubation rate, insertion time, observation time, adenoma detection rate (ADR), and polyp detection rate (PDR).

### Definitions

BBPS addresses three individual colonic segments: the right colon, transverse colon, and left colon. It assesses bowel preparation during withdrawal of the colonoscope. Each segment of the colon was scored from 0 to 3, with higher scores indicating superior cleansing, and summed for a total score ranging from 0 to 9. A score of ≥ 6 points was considered to represent good bowel preparation, and a score of < 6 points indicated poor bowel preparation.

Insertion time was defined as the time from passing of the scope through the anus until it reached the cecum. Observation time was defined as the time from cecal intubation until the end of the procedure, excluding polyp observation time and treatment time. ADR was defined as the proportion of procedures in which at least one adenoma was detected, and the PDR was defined as the proportion of procedures in which at least one polyp was detected. The tolerability of PEG and macrogol 4000 as colon cleansers was also assessed using a questionnaire. The patients were asked to rate each medication on the following four-point scale: A, extremely unpleasant to take, never want to take it again; B, unpleasant to take, but would take again for colonoscopy; C, slightly unpleasant to take, but within the limits of tolerability; or D, easy to take. Responses to C and D were defined as “well tolerated.”

### Sample size calculation and statistical analysis

At the start of this study, there had been no report of the use of macrogol 4000 as an adjuvant colon cleanser for colonoscopy. In a previous study, the use of PEG helped in achieving adequate bowel cleanliness in 56% of patients with chronic constipation [18]. In our hospital, between January and August 2019, we used picosulfate the night before colonoscopy, followed by PEG 1500 mL on the morning of the procedure as colon cleansers for patients with chronic constipation, of whom 75% achieved a BPPS score of ≥ 6 points, indicating good bowel cleanliness. With the expected proportion of good bowel cleanliness of 75%, a threshold value of 55% good bowel cleanliness deemed unacceptable, *α* = 0.1, and 1 − *β* = 0.8, 35 patients were required for the study. Assuming a dropout rate of 10%, we considered that a total of 40 patients would be needed. All continuous variables were expressed as medians. EZR (version 1.27; Saitama Medical Center, Jichi Medical University, Japan) [18] was used for all statistical analyses.

## Results

### Patient baseline characteristics

Between November 2019 and July 2020, 1642 patients underwent colonoscopy screening at our hospital. Of these, 43 patients fulfilled all the inclusion and exclusion criteria and provided informed consent. Two patients failed to present for the procedure after providing consent. One patient was unable to ingest 1500 mL of PEG and was excluded from the study. The remaining 40 patients were included in the study. Table 1 shows the baseline characteristics of the patients. The median age was 64 years (range 29–84 years), and 52.5% were men and 47.5% were women. The median height was 161 cm (142–178 cm), and the median weight was 60 kg (range 37–88 kg). The performance status was 0 in 38 patients (95%) and 1 in two patients (5%). None of the patients were taking tricyclic antidepressants. Thirty-eight patients (75.0%) had used laxatives, and seven of them (17.5%) used stimulant laxatives. The proportion of patients with severe constipation, which was defined as the regular use of stimulant laxatives or the passage of fewer than two bowel movements per week, was 17.5% (7/40). Twenty cases (50%) underwent colonoscopy for the first time, and another 20 cases had colonoscopy before.

**Table 1:** Patient baseline characteristics (n = 40)

Characteristic	Value
Age, (range)	64 (29–84)
Male/female, n (%)	21 (52.5)/19 (47.5)
Performance status	
0/1/2–4, n (%)	38 (95.0)/2 (5.0)/0 (0)
Height, cm (range)	161 (142–178)
Weight, kg (range)	60 (37–88)
Comorbidity	
Hypertension, n (%)	12 (30.0)
Hyperlipidemia, n (%)	10 (25.0)
Diabetes, n (%)	5 (12.5)
Chronic kidney disease, n (%)	4 (10.0)
Prescription of antidepressants, n (%)	0 (0)
Prescription of laxative, n (%)	26 (65.0)
Prescription of stimulant laxatives, n (%)	7 (17.5)
Severe constipation, n (%)	7 (17.5)
Previous colonoscopy	
For the first time/repeated, n (%)	20 (50.0)/20 (50.0)

### Effectiveness of colonic cleansing

**Fig. 1:**
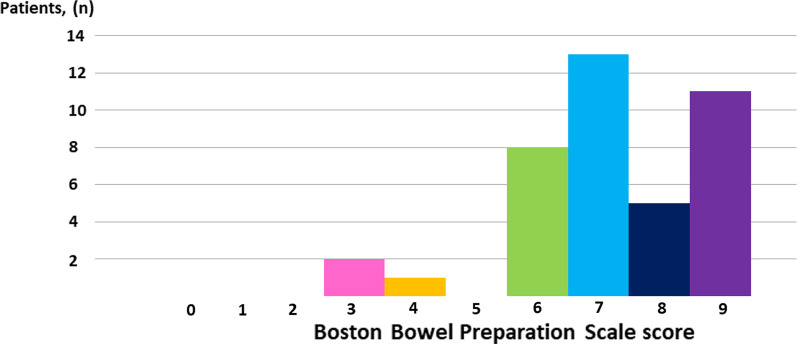
Distribution of total Boston bowel preparation scale scores in this study

**Fig. 2:**
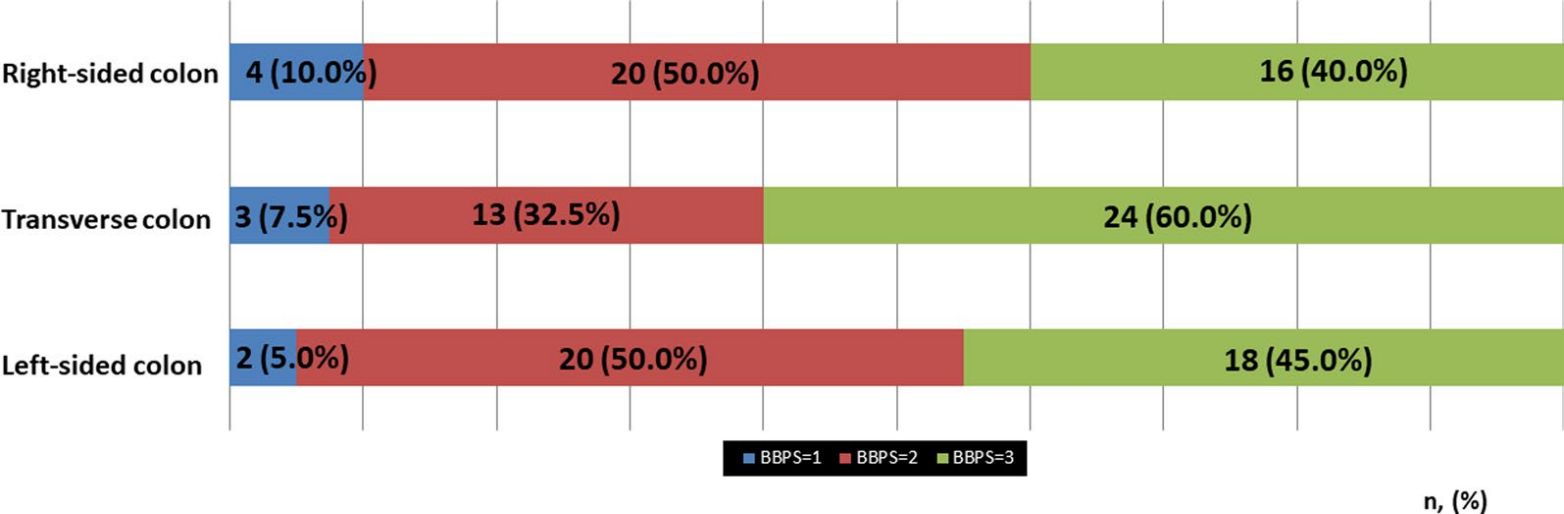
Boston bowel preparation scale (BBPS) score of each segmental colon. Blue area indicates patients with BBPS 1 point; red area indicates patients with BBPS 2 points; green area indicates patients with BBPS 3 points

The distribution of the BBPS scores is shown in Fig. 1. The median BBPS score was 7 (range 3–9). The BBPS score was ≥ 6 points in 37 patients (92.5%). Figure 2 and Table 2 show the BBPS scores of each segmental colon. In the right-sided colon, the BBPS score was 1 in 4 patients (10.0%); 2, 20 patients (50.0%); 3, 16 patients (40%). In the transverse colon, the BBPS score was 1 in 3 patients (7.5%), 2 in 13 patients (32.5%), and 3 in 24 patients (60.0%). In the left-sided colon, the BBPS score was 1 in 2 patients (5.0%), 2 in 20 patients (50.0%), and 3 in 18 patients (45.0%).

**Table 2:** Effectiveness of colonic cleansing

n = 40	Value
Total Boston bowel preparation scale (range)	7 (3–9)
Boston bowel preparation scale ≥ 6, n (%)	32 (92.5)
Right-sided colon	
0/1/2/3, n (%)	0 (0)/4 (10.0)/20 (50.0)/16 (40.0)
Transverse colon, n (%)	
0/1/2/3, n (%)	0 (0)/3 (7.5)/13 (32.5)/24 (60.0)
Left-sided colon	
0/1/2/3, n (%)	0 (0)/2 (5.0)/20 (50.0)/18 (45.0)

### Bowel preparation or endoscopy-related outcomes

As shown in Table 3, the median volume of total PEG taken was 1500 mL (range 1500–2000 mL), and the median volume of PEG taken until defecation was clear was 1500 mL (range 1000–2000 mL). The median number of defecations before the procedure could be started was 7 (range 3–20). The median time required was 210 min (90–360 min). In 26 cases, colonoscopy could be started when after the administration of a PEG dose of 1500 mL or less, while 14 cases required additional PEG, and in one case, clean stool was not achieved even after taking 2000 mL of PEG. Cecal intubation was achieved in all cases, the median insertion time was 6.0 min (2.3–22 min), and the median observation time was 8.8 min (4.0–16.0 min). The ADR and PDR were 60.0% and 75.0%, respectively. One patient was unable to ingest 1500 mL of PEG due to mild nausea and was excluded from the study. No other side effects, such as abdominal pain or nausea, were observed for either macrogol 4000 or PEG.

**Table 3:** Bowel preparation and endoscopic clinical outcomes

n = 40	Value
PEG taken	
Total, mL (range)	1500 (1500–2000)
Until defecation was clear, mL (range)	1500 (1000–2000)
Time, min (range)	210 (90–360)
Defecations, n	7 (3–20)
Additional dose, n (%)	14 (35.0%)
Adverse events, n (%)	0 (0%)
Cecal intubation	40 (100%)
Insertion time, min (range)	6.0 (2.3–22.0)
Observational time, min (range)	8.8 (4.0–16.0)
ADR, n (%)	24 (60.0%)
PDR, n (%)	30 (75.0%)

*PEG* polyethylene glycol, *ADR* adenoma detection rate, *PDR* polyp detection rate

### The tolerability of macrogol 4000 and PEG

**Fig. 3:**
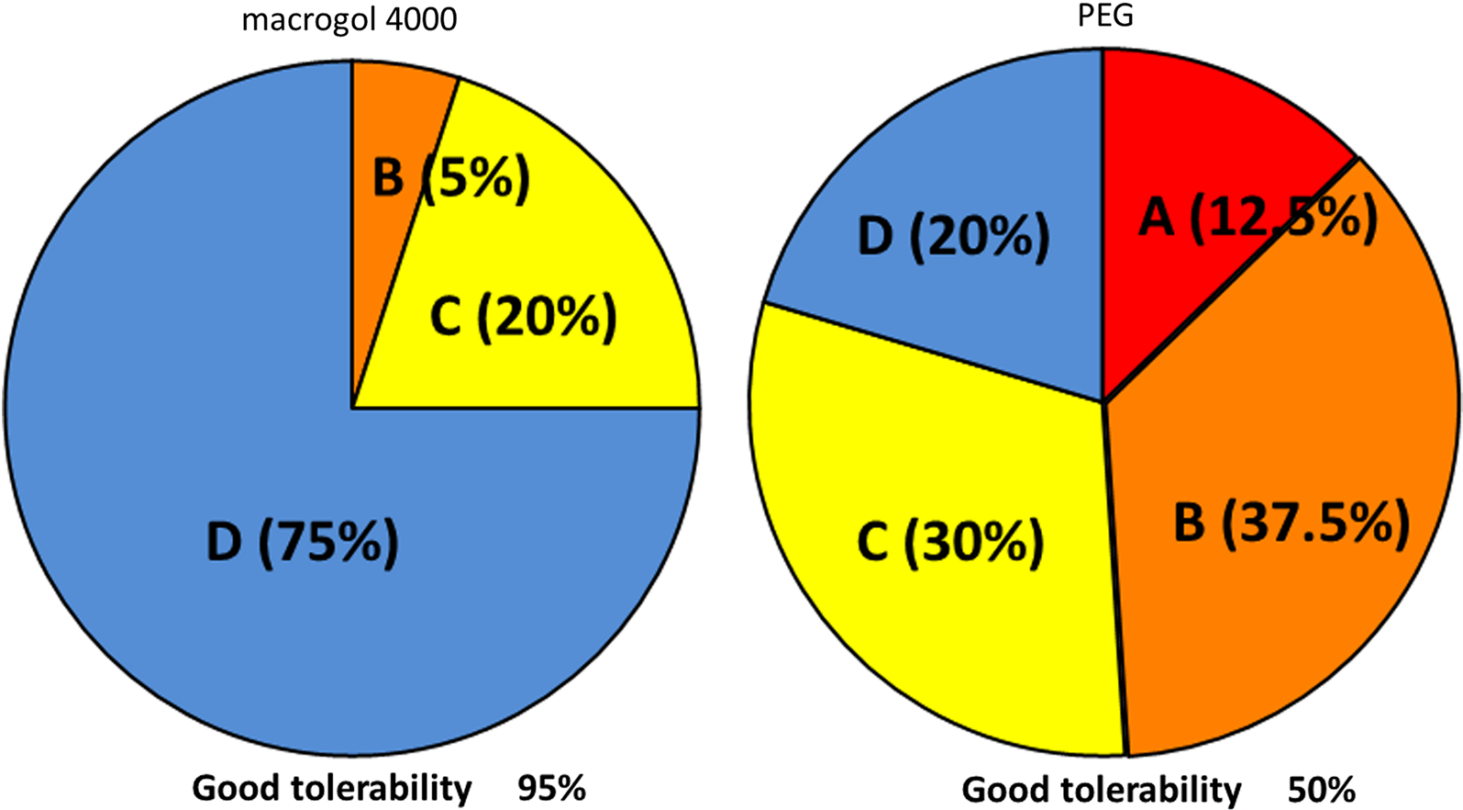
Tolerability of polyethylene glycol (PEG) and macrogol 4000. **a** Extremely unpleasant to take, never want to take it again; **b** unpleasant to take, but would take again for colonoscopy; **c** slightly unpleasant to take, but within the limits of tolerability; **d** easy to take; proportion of (**c**) and (**d**) defined as good tolerability

As shown in Fig. 3, the tolerability assessments of the colon cleansers according to the four-point scale (A, extremely unpleasant to take, never want to take it again; B, unpleasant to take, but would take again for colonoscopy; C, slightly unpleasant to take, but within the limits of tolerability; or D, easy to take) were as follows: Macrogol 4000: A, 0 patients (0%); B, 2 patients (5.0%); C, 8 patients (20%); D, 30 patients (75%) PEG: A, 5 patients (12.5%); B, 15 patients (37.5%); C, 12 patients (30.0%); D, 8 patients (20.0%). A proportion of patients rated the tolerability of macrogol 4000 and PEG as 95.0% and 50.0%, respectively.

## Discussion

To the best of our knowledge, this is the first prospective study to investigate the efficacy and safety of macrogol 4000 intake the night before colonoscopy, in addition to the PEG dose of 1500–2000 mL for colonoscopy preparation, in patients with chronic constipation. The results showed that bowel preparation was good in 92.5% of cases, indicating the effectiveness of this method. There were almost no adverse events, and it was well tolerated. We evaluated the degree of bowel preparation for each colon. The BBPS was better at the transverse colon than at the right-sided and left-sided colon. We considered some potential reasons for this result. In our study, eight patients had diverticula in the sigmoid or ascending colon, while there were no diverticula in the transverse colon. Since stool is clogged in the diverticulum, it is considered to be one of the causes of a worsening of BBPS values in the right- or left-sided colon. Another reason is that we withdrew the colonoscope and observed the whole colon in the supine position. In this position, stool and liquid more likely pooled in the ascending or descending-sigmoid colon, potentially worsening BBPS values in the right- or left-sided colon.

Polypectomy can prevent the onset of colorectal cancer. However, if bowel preparation is poor, polyps < 10 mm in size are particularly difficult to detect, and the ADR declines [4]. One study found that compared with an ADR of > 20%, an ADR < 20% increased the risk of interval cancer by tenfold [19]. Another study reported that a 1% improvement in the ADR led to a 3% reduction in the development of colorectal cancer and a 5% reduction in colorectal cancer deaths [20]. Colonoscopy results from this study were extremely good, with an ADR of 60.0%, PDR of 75.0%, and median insertion time of 6.0 min, thus indicating that this method was also effective from the standpoint of colonoscopy. The potential reasons of relatively higher ADR and PDR were, first, the very good bowel preparation, and second, that all colonoscopies were performed by an endoscopist, who was certified by the Japanese Gastroenterological Endoscopy Society.

Although one patient was unable to ingest 1500 mL of PEG, intake of macrogol 4000 did not cause any adverse events, such as abdominal pain or vomiting. Previous studies have reported the occurrence of abdominal pain, diarrhea, nausea, and other adverse events in 4.5–15.7% of patients who received macrogol 4000 [16, 21, 22]. However, this might be attributed to the continual intake of macrogol 4000 for at least a week. In contrast, in our study, macrogol 4000 was ingested only once on the night before colonoscopy. We found that even though macrogol 4000 has same composition as PEG, dissolving it in apple juice minimized the discomfort associated with its use.

In Japan, the dose of PEG is generally lower than that used in Western countries. Nevertheless, efforts to improve the effectiveness of colon cleansing and to reduce this dose are required. Conventionally, stimulant laxatives have been used in Japan, such as sodium picosulfate hydrate, senna, and bisacodyl. However, the use of these stimulants for colon cleansing has been reported to cause ischemic colitis and requires careful attention [23, 24]. Macrogol 4000 is a non-stimulant laxative that is unlikely to overstimulate the bowel and can, thus, be safely used for colon cleansing. Several previous reports have described the use of additional non-stimulant laxatives the previous night as adjuvant colon cleansers. In one randomized controlled trial, lubiprostone was shown to significantly improve the BBPS score (7.44 + 0.14 vs. 6.36 + 0.16, *p* < 0.0001) when compared with the placebo [25]. Another study of mosapride as an adjuvant to bowel preparation with 2000 mL of PEG found that the addition of mosapride to 2000 mL of PEG resulted in significantly better bowel preparation in the left colon (78.2% vs. 65.6%, *p* < 0.05) [26]. When mosapride was used as an adjuvant for bowel preparation with 2000 mL or 1500 mL of PEG, the results for 1500 mL were not inferior to those obtained with 2000 mL, while the 1500 mL dose was better tolerated [27]. These findings suggested that the use of mosapride may reduce the volume of PEG ingested. Yoshida et al*.* reported that the continuous intake of macrogol 4000 for 1 week prior to colonoscopy improved the rate of effective bowel preparation to 72.6%, in addition to the improvement in insertion time and discomfort during insertion; however, this was a retrospective study [21]. In this study, we evaluated intake of macrogol 4000 only on the night before the colonoscopy procedure to ensure patient adherence. Hence, future comparative studies are required to investigate whether taking additional medication only on the night before the procedure is sufficient or whether it should be taken for approximately a week, taking into account both patient adherence and efficacy.

One of the major strengths of our study is that it was the first prospective study on the use of macrogol 4000 for colon bowel preparation. Our results not only support the findings of previous retrospective studies of its value, but we also observed almost no side effects. In addition, this study only included patients with constipation.

This study has several limitations. First, it was conducted at a single center. In addition, it was a single-arm, non-randomized trial. A comparative study, such as a randomized control trial would be ideal to evaluate the effect of macrogol 4000. However, since there has been no prospective study of macrogol 4000 as a bowel preparation in addition to PEG so far, conducting a comparative study would involve ethical issues. Therefore, we first evaluated the efficacy of an exploratory single-arm study to perform an appropriate randomized control study in the future. Second, the study included only Japanese patients, and the results may not be applicable to patients from other regions who have greater mean body weight than that of Japanese patients. Westerners and Asians have different physiques, and studies have also suggested that there may be racial differences in the efficacy of PEG [28], suggesting that complex elements may have some effect. Third, only those who could take 1500 mL PEG were evaluated in this study; the effect of macrogol 4000 in patients who could not take 1500 mL PEG still remains unknown.

When conducting colonoscopies, the choice of bowel cleansing agents is extremely important. The results of this study suggest that macrogol 4000 as an adjuvant bowel preparation may be used as a new alternative. However, it is too early to draw concrete conclusions and further studies with larger number of patients are required.

## Conclusion

The use of macrogol 4000 in addition to PEG before colonoscopy is an effective and safe bowel preparation method for patients with chronic constipation.

## Data Availability

The data can be provided by the corresponding author on reasonable request.

## Abbreviations

PEG: Polyethylene glycol
BBPS: Boston bowel preparation scale
ADR: Adenoma detection rate
PDR: Polyp detection rate
